# F154. ABERRANT SALIENCE NETWORK FUNCTIONAL CONNECTIVITY IN AUDITORY VERBAL HALLUCINATIONS: A FIRST EPISODE PSYCHOSIS SAMPLE

**DOI:** 10.1093/schbul/sby017.685

**Published:** 2018-04-01

**Authors:** Paris Alexandros Lalousis, Pavan Mallikarjun, Thomas Frederick Dunne, Kareen Heinze, Renate Reniers, Matthew R Broome, Baldeep Farmah, Femi Oyebode, Stephen Wood, Rachel Upthegrove

**Affiliations:** 1 University of Birmingham; 2 University of Oxford; 3 Forward Thinking Birmingham; 4 Orygen, the National Centre of Excellence in Youth Mental Health

## Abstract

**Background:**

Auditory verbal hallucinations (AVH) often lead to distress and functional disability, and are frequently associated with psychotic illness. Theories of abnormal integration have been proposed to explain symptoms of schizophrenia, including delusions and hallucinations, with a central abnormality being aberrant activity in intrinsic brain networks such as the default mode network (DMN) or the salience network (SN). Previous investigations of patients with schizophrenia assessing functional connectivity (FC) have used a seed-based functional connectivity approach (sb-FC), with seed placement in brain areas responsible for auditory processing, language, and memory; the striatum, and in areas of DMN. These have generated some conflicting results, possibly because of the varying seed placement. The aim of the current study was to address these confounding factors by investigating the intrinsic FC in first episode psychosis (FEP) patients with AVH using within-sample AVH symptom capture seeds. It was hypothesised that patients would show aberrant resting state FC between areas of the DMN and SN and these areas.

**Methods:**

Eighteen FEP individuals and 20 healthy controls were recruited. All the participants underwent resting-state functional Magnetic Resonance Imaging (rs-fMRI). The Data Processing Assistant for Resting-State fMRI Advanced Edition (DPARSFA) V3.1 (http://rfmri.org/DPARSF) (Yan & Zang, 2010) and the statistical parametric mapping software 8 (SPM8) (SPM, Friston, The Wellcome Department of Cognitive Neurology, London, Uk; http://www.fil.ion.ucl.ac.uk/spm) were used to preprocess and analyze the data.

**Results:**

Patients showed increased FC between left insula and bilateral cerebellum, and angular gyrus; and increased FC between left claustrum and left cerebellum and postcentral gyrus. There was reduced FC in FEP patients with AVH between left claustrum and left insula compared to HC. The FC between left insula and left claustrum seeds for patients and HC is shown separately in supplementary information. There were no significant correlations between DUP, dose of antipsychotic medications, and severity of hallucinations and the mean coefficients of clusters that were significantly different between FEP patients and HC.

**Discussion:**

FEP patients showed increased functional connectivity between left insula and bilateral cerebellum and angular gyrus; and increased functional connectivity between left claustrum and left cerebellum and postcentral gyrus. We also found reduced functional connectivity between left claustrum and left insula in FEP patients compared to HC. It is possible the pathology of AVH is primarily located in the insula and angular gyrus. However, given our results of both the left insula seed in patients and HC shows connectivity with right insula and anterior cingulate cortex (key regions of SN) and literature from patients with chronic AVH, the suggestion may be that resting state dysconnectivity within the DMN and SN are implicated in the generation of AVH, which during the experience itself will further involve temporal and auditory networks. Furthermore, decreased intrinsic functional connectivity between the claustrum and the insula may lead to compensatory over activity in parts of the auditory network including areas involved in DMN, auditory processing, language and memory, leading to the complex and individual content of AVH when they occur.

